# Risk perception of Ebola virus disease and COVID-19 among transport drivers living in Ugandan border districts

**DOI:** 10.3389/fpubh.2023.1123330

**Published:** 2023-06-15

**Authors:** María José Blanco-Penedo, Hannah Brindle, Megan Schmidt-Sane, Alex Bowmer, Constance Iradukunda, Herbert Mfitundinda, Jude Rwemisisi, Grace Nicholas Mukiibi, Christine Fricke, Simone Carter, David Kaawa-Mafigiri, Shelley Lees

**Affiliations:** ^1^Faculty of Infectious and Tropical Diseases, London School of Hygiene and Tropical Medicine, London, United Kingdom; ^2^Department of Clinical Research, Faculty of Infectious and Tropical Diseases, London School of Hygiene and Tropical Medicine, London, United Kingdom; ^3^Institute of Development Studies, University of Sussex, Brighton, United Kingdom; ^4^Department of Global Health and Development, London School of Hygiene and Tropical Medicine, University of London, London, United Kingdom; ^5^Department of Social Work and Social Administration, School of Social Sciences, College of Humanities and Social Sciences, Makerere University, Kampala, Uganda; ^6^Baylor Uganda, Kampala, Uganda; ^7^Department of Anthropology and African Studies, Johannes Gutenberg University, Mainz, Germany; ^8^Public Health Emergencies, UNICEF, Kinshasa, Democratic Republic of Congo

**Keywords:** Ebola, COVID-19, risk, behavior, drivers, border, movements, policy

## Abstract

**Background:**

Cross-border movements between districts bordering Uganda and the Democratic Republic of Congo (DRC) are common due to the interdependence between populations on either side, though this increases the risk of the international spread of infectious diseases. Due to the nature of their work, boda boda drivers (motorcycle taxis), taxis and truck drivers continue to cross the border during epidemics. However, perceived risk of contracting and spreading communicable diseases may be influenced by several factors such as the level of education, packaging and perception of health care messages, limited interaction with local socio-cultural dynamics or personal experiences. This study aims to explore differences in movement patterns and risk perceptions as factors for transmission among transport drivers in Ugandan border districts during the 2018–2020 Ebola Virus Disease (EVD) epidemic and the current COVID-19 pandemic.

**Methods:**

Between May and June 2021, in-depth interviews and focus group discussions were conducted with transport drivers in three Ugandan districts bordering DRC (Kasese, Kisoro and Hoima). Participants were asked about their knowledge and beliefs about EVD and COVID-19, perceived risk during epidemics, reasons for, and travel patterns during the EVD epidemic and COVID- 19 pandemic. A thematic content analysis was applied.

**Results:**

Participants’ awareness of EVD was higher than that of COVID-19 however, the risk of transmission of Ebola virus was perceived as a remote threat. Measures restricting mobility during the COVID-19 pandemic had a greater impact on transport drivers compared to those implemented during the EVD epidemic, and were perceived as prohibitive rather than protective, largely due to fear of reprisals by security officers. Despite this, drivers were unlikely to be able to comply with the restrictions as they relied on their work as a source of income.

**Conclusion:**

The vulnerabilities of transport drivers should be considered in the context of epidemics such EVD and COVID-19 in Uganda. Policy makers should address these particularities and assess the impact of public health measures on transport drivers’ mobility and involve them in designing of mobility-relatedpolicies.

## 1. Introduction

Cross-border movements are essential in East Africa for socioeconomic and community connectivity. Activities such as trade, visiting families, or tourism can increase the risk of transmission of diseases to new areas (1). During the Ebola virus disease (EVD) epidemic in West Africa in 2014–2016, the virus spread from Guinea to Sierra Leone and Liberia through travel across borders (2). Many borders in Africa were arbitrarily imposed during the colonial era and did not respect the social or economic relations between the previously existing communities (3). The Ugandan/Democratic Republic of the Congo (DRC) border was delimited by colonial administrators in the late 19th and early 20th centuries (4). This administrative boundary is highly porous and informal border crossings are common, sometimes used to facilitate smuggling, to avoid paperwork, “ad hoc taxes,” waiting times, or restrictions (5, 6). These multiple and diversified interactions make bordering districts distinct entities “where people mix and move freely, often with family, friends and business contacts on both sides” (7).

The DRC declared its 10th outbreak of EVD on 1 August 2018 in North Kivu province (8). On 25 June 2020, the epidemic was declared over with a total of 3,481 cases and 2,299 deaths reported, becoming the second-largest outbreak in history, and the largest in the country to date (9). North Kivu, situated in eastern DRC and bordering Uganda, is a long-standing conflict zone, with fragile political stability and frequent movements of the population (10). Shortly after notification of the outbreak, the Ugandan Ministry of Health (MoH) activated the Public Health Emergency Operation Centre (PHEOC) and the EVD National Task Force with Uganda-DRC bordering districts classified as a high priority to detect and respond to EVD cases. Measures implemented included community-based surveillance, EVD treatment centers, reinforcement of screening and infection prevention and control (IPC) measures at points of entry (PoEs), and vaccination of healthcare workers (11, 12). In total, there were three cases of EVD reported in Uganda during the epidemic after crossing the border from DRC (13).

By March 2019, the Infectious Disease Institute of Uganda (IDI), on behalf of the Ugandan MoH, assessed population movement patterns using the PopCAB toolkit (14). Changes in the typical patterns of movement and use of new ground crossings between Uganda, DRC and Rwanda were identified despite border restrictions. The results were used by the Uganda National Task Force to increase surveillance, infection prevention and control risk communication strategies in areas at risk for the importation of EVD, and collaborating with community-level sectors that interact with populations connected to these areas (11).

More recently, on 20th September 2022, the Uganda MoH declared another outbreak of EVD due to the Sudan ebolavirus species [Sudan ebolavirus disease (SVD)] in the district of Mubende, located in central Uganda, 130 km from the capital, Kampala. However, this is the first time in more than a decade that SVD has been reported in the country and the epidemic is ongoing with signs of ending soon (15).

In relation to the spread of epidemics and Uganda’s governmental response, as of 8th December 2022, Uganda has reported a total of 169,715 cases of COVID-19 (16). At the start of the COVID-19 pandemic, from March until June 2020 Uganda implemented strict control measures, including the restriction of movements at PoEs and the closure of borders (17). However, due to the connectivity of the border region, many continued to travel across informal PoEs (panya routes) though trade severely decreased overall. All Ugandan international bordering districts were classified as high-risk for the emergence and spread of COVID-19 (18). Since July 2022, measures have been adapted according to increased technical knowledge, the epidemiological situation in the country and the impact of the pandemic (19).

Several studies show that individual factors such as age, gender or occupation influence travel behaviors and movement patterns (20–23). Poletti and colleagues argued that individuals’ risk perception is based on the memory mechanism, and that risk perception during epidemics is initially overestimated (24).

In many contexts, the concept of risk behaviors is linked to a lack of knowledge or awareness about the risk of infection or transmission. However, a growing number of studies reflect the complexity and dynamics of people’s risk-taking behavior (25).

Truck drivers have historically been identified as a “risk group” for the transmission of infectious diseases. They interact with a large social network, traveling through different communities on their journeys, thus increasing the risk of contagion and spread of some infections (26). Previous research has shown that during the EVD epidemic, Ugandan truck drivers continued to cross the border with DRC despite their concern about becoming infected (7). Additionally, in Uganda, truck drivers were identified as one of the first high-risk groups for the international and domestic transmission of COVID-19 (27). Since the beginning of the COVID-19 pandemic, strategic recommendations and guidance for truck drivers have been developed by the East and Southern Africa Health emergency group from the World Health Organization Regional Office for Africa (28) and the East African Community Secretariat (29), including the need for pre-departure testing, evidence of a negative test certificate or limiting the number of passengers. With the development of COVID-19 vaccines, some of these requirements have been replaced by vaccination certificates (30).

Boda boda drivers, the name attributed to motorcycle taxis in Uganda, is derived from their use in crossing international borders between Uganda and Kenya, including through unauthorized points and for smuggling purposes (31). The low cost and adaptability to the poor road infrastructure has made boda bodas one of the preferred means of transport in Uganda (32). Boda boda drivers, also called boda boda men, carry cargo or passengers on the back of their motorbikes, and therefore, they have also been identified as a key group in the transmission of pathogens, including EVD (6, 33).

Unlike boda boda drivers, taxi drivers have established routes, although some have so-called “special” services with flexible routes. During the current COVID-19 pandemic, non-compliance with mobility measures and movements through unauthorized border points by taxi drivers have been reported (34, 35).

In the light of the risk of disease transmission and vulnerability of transport drivers, we conducted a qualitative study to explore the internal and cross-border movement patterns, motivations, and perceptions of risk of Ugandan transport drivers during the 2018–2020 Ebola epidemic and the start of the COVID-19 pandemic.

## 2. Methods

The findings presented in this paper are drawn from data collected as part of a larger mixed-methods study “Building trust and community ownership of Ebola and Community Engagement in DRC/Uganda border communities” which comprised five domains including COVID-19 communication, health seeking behaviors during outbreaks, trust in formal and informal authorities, movements, and human-wild-life conflict.

Data collection took place in three Ugandan districts bordering DRC, Kasese, Kisoro and Hoima from 1-30^th^ May 2021. Participants for the movement domain were recruited using a combination of snowball and purposive sampling to ensure the sample comprised a mixture of genders, age groups, and occupations. We purposely sampled for “higher-risk” occupations like transportation workers, including long distance truck drivers, taxi drivers (minibus taxi and four-seater taxis), and boda boda drivers.

Participants provided informed written consent to take part in either in-depth interviews (IDIs) or focus group discussions (FGDs). The objective of the IDIs was to address in depth sensitive aspects, which are potentially more challenging to discuss in a group session. However, FGDs were intended to stimulate and generate questions, discussions and new ideas. IDIs covered a range of topics including demographics, work history, beliefs about and perceived risk of EVD and COVID-19, trust in various actors and health-seeking behaviors. FGDs covered, additionally, movement patterns. In this study, risk refers to behavioral practices related to structural and social factors, rather than to individual exposure decisions. One hundred and thirty-nine IDIs and FGDs were carried by trained research assistants and researchers of the original study. Identifiable participants’ information was completed in a separate form and linked to the activity via a unique identifier. IDIs and FGDs were carried out in Rufumbira for Kisoro, Runyoro for Hoima and Rukhonzo for Kasese and once completed, translation into English was done by the research assistants.

FGDs and IDIs were recorded using the Open Data Kit (ODK) Collect application on Android phones (36). The recordings were encrypted on saving and synchronized to the ODK central server hosted at the London School of Hygiene and Tropical Medicine (LSHTM) via Wi-Fi or 4G. Demographic data about the participants were collected using separate ODK Collect forms. The audio files were downloaded from the ODK central server for transcription and translation.

Data were classified according to type of data (IDIs or FGDs), district of residence and occupation (boda boda, taxi and truck drivers). Thematic content analysis was undertaken by generating initial codes and themes based on techniques described by Ryan and Bernard (37). This included the use of inductive (searching for repetitions, similarities, and differences, and cutting and sorting) and deductive (following the topic guide, finding key words in the text, and searching for missing data) approaches. Data were coded using the NVivo 12 software.

The themes were reviewed according to the research question and contrasted with codes generated by the researchers. The different themes have been gathered into two general topics focusing on risk- related factors in the mobility and movements of transport drivers. These two general themes are granulated into themes and sub-themes to achieve a more in-depth analysis of the mobility of transport drivers during the EVD and COVID-19 epidemics.

## 3. Results

A total of 26 drivers participated in 11 IDIs and three FGDs. All participants were male and their specific occupation, district, and location where the interview or FGDs took place are depicted in Table 1.

**Table 1:** IDIs/FGDs participants by occupation, district, and place of data collection.

Occupation	District	Location
FGDs (5 participants in each)		
Boda boda drivers	Hoima	Hoima city
Truck drivers	Kasese	Hima town council
Boda boda drivers	Kisoro	Bunagana border
IDIs		
Boda boda driver	Hoima	Hoima city
Boda boda driver	Hoima	Kaiso town
Boda boda driver	Kasese	Bwera customs
Boda boda driver	Kasese	Hoima town
Boda boda driver	Kisoro	Kisoro town
Boda boda driver	Kisoro	Kyanika town
Taxi Driver	Hoima	Hoima city
Taxi Driver	Kisoro	Kisoro town
Truck Driver	Kisoro	Bunagana border

The first main theme focused specifically on how EVD and COVID-19 influenced the transport drivers’ movements. This included their destination, their health, the health of the passengers and whether they were carrying dead bodies.

The second overarching theme related to the perception of risk and how this influenced the mobility of the transport drivers during the EVD epidemic and COVID-19 pandemic. Prevalent factors (associated with occupation and place) and factors associated with EVD, and COVID-19 (diseases’ awareness, trust, and fear) were also covered under this theme.

**Figure 1:**
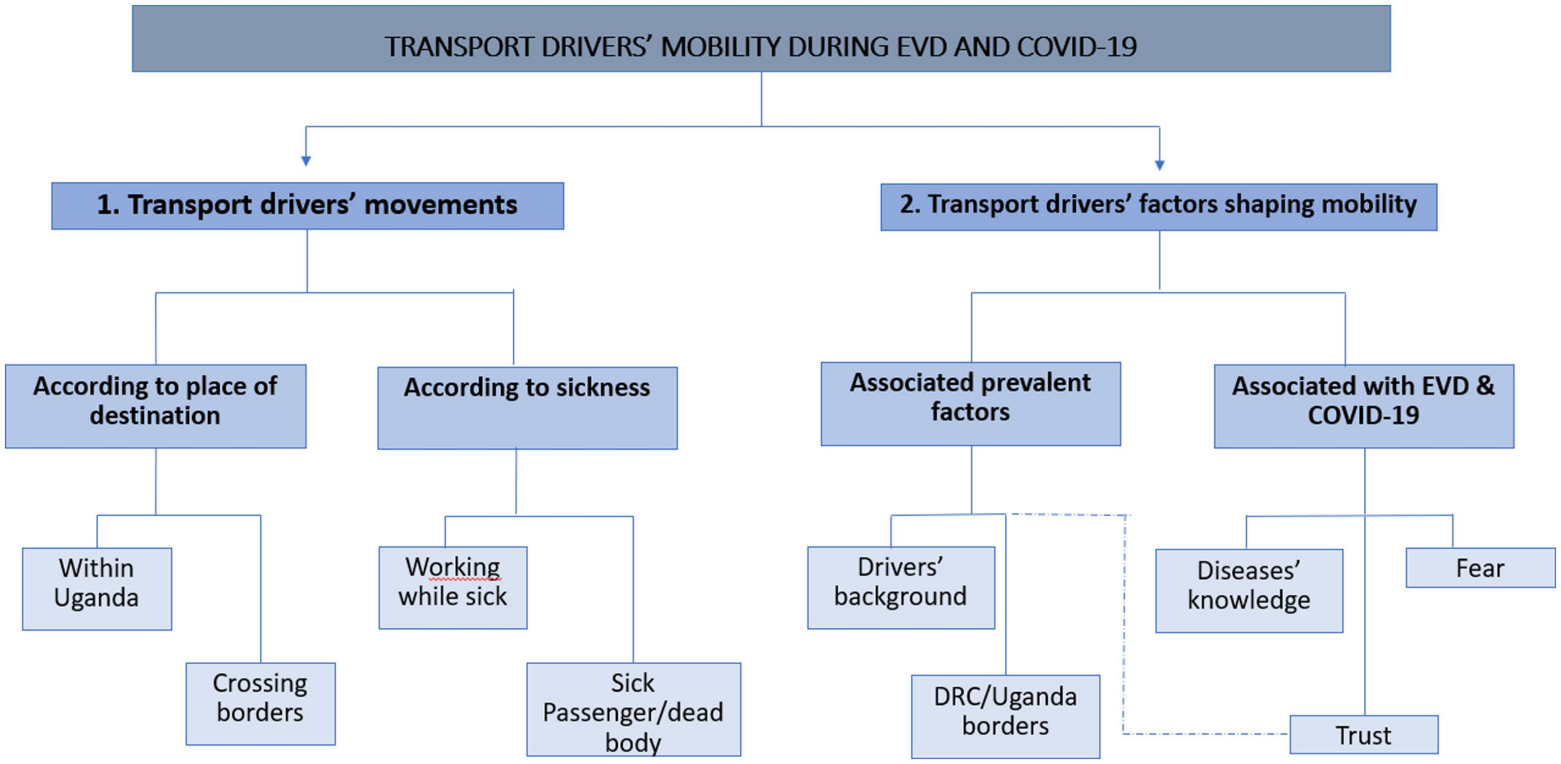
Organigram for themes classification.

Organigram for themes classification is depicted in Figure 1.

### 3.1. Transport drivers’ movements

This first theme aims to explore the activity of drivers during the EVD epidemic and the COVID-19 pandemic, and how this would be affected by drivers living in border districts. It also explores how it would affect whether they themselves or the person being transported would be symptomatic, including the transport of dead bodies.

#### 3.1.1. According to place of destination

##### 3.1.1.1. Within Uganda

Ugandan mobility regulations during COVID-19, such as curfews, particularly affected taxi drivers and boda boda drivers who experienced a reduction in their working hours. Moreover, restrictions on gatherings or the use of public transport resulted in few or no passengers with drivers having to resort to transporting cargo. For most drivers, their income depends directly on the number of trips made, so the movement restrictions had a strong impact on their economies and those of their families.

*“We were being told to only transport cargo but not passengers, so we used to come to town, and you look for what to carry, and our curfew was beginning at 2 pm, if you do not find anyone giving you cargo to carry then you would go back empty-handed, so these pushed us back.” (Boda boda driver, Hoima)*.

Regulations were experienced as restrictions by security forces rather than preventive measures to protect them. There is no reference in the drivers’ narrative to the fact that these measures could mitigate the risk of infection or spread of the virus or that the perception of risk once implemented would be reduced. Rather, they focus on the administrative-legal consequences of non-compliance.

*“The time of lockdown was terrible because we never used to work and at times were forbidden to move. They get you gathered and have you arrested; they got you driving, they just arrest you.” (Taxi driver, Hoima).*

Unlike the movement restrictions during COVID-19, no mobility reduction measures were put in place in Uganda during the EVD epidemic and therefore did not have a negative impact on drivers’ economies.

##### 3.1.1.2. Crossing borders and associated risks

Regarding movements across borders between the two countries, there were restrictions on mobility during both epidemics. However, perceptions of border measures were different in EVD and COVID- 19. When drivers talked about EVD, they often mentioned a medical screening team to identify and refer suspect cases to isolation facilities. After passing the checks and usual procedures, they could move without further restrictions. One of the participants explained the measures that the government and its partners had put in place to stop the EVD spread:

*“As you come closer to the border, they brought some tents. I will call them the check points. They put there something to wash our hands and the gun temperatures, the sanitizers. They were there at the check points.” (Boda boda driver, Kasese).*

When talking about the inability to cross the border during COVID-19, drivers did not refer to the risk of disease transmission, but rather the administrative and legal consequences. When asked about the existence of porous borders for crossing into DRC beyond the official entry points, one of the boda boda argued the reasons why these unofficial points were used:

*“Yes, the UPDF officer (Uganda People’s Defense Force) is a sanitizer but also a person, who has a stomach which needs to eat, so it is between you and him to negotiate, if he agrees you cross. (Boda boda driver, Kasese).”*

Officers were accused of asking for bribes to allow passage or, in the case of those suspected of having COVID-19, to avoid referral to a checkpoint clinic. Again, these restrictions, imposed during the COVID-19 pandemic, hampered drivers’ mobility and consequently their fragile economy, sometimes leading them to seek alternative routes via unauthorized routes.

*“In times of Ebola, we used to cross to Congo with our motorcycles and then come back, but with covid outbreak, we cannot cross to Congo with our motorcycles. We illegally use shortcuts to Congo.” (Boda boda driver, Hoima).*

Throughout this theme, a greater acceptance of the restrictions imposed during the EVD epidemic is observed, as it affected the mobility of transport drivers to a lesser extent and allowed them to continue to carry out their economic activity.

#### 3.1.2. Movements according to sickness

##### 3.1.2.1. Working while sick

In contrast to the impact of the restrictions imposed during EVD and COVID-19 on their movements, we found very few references in the drivers’ discourse to the impact of being ill on their work. It is barely mentioned that they restrict their activity without further argument, thus suggesting a minor impact on their activities. In one of the interviews, the facilitator asked one of the drivers if he would continue working if he had a headache or fever.

*“I just keep at home.” (Taxi driver, Hoima).*

##### 3.2.2.2. Carrying sick people/death bodies

The situation is reversed when it comes to the transport of people who may present with symptoms of COVID-19 or EVD, when drivers’ responses are accompanied by an argument to justify their decisions. Thus, some of the drivers argue that they are interested in the type of symptomatology to ensure that they do not suffer from EVD or COVID-19. For some, the need to earn an income and the desire to help people in their community is sufficiently relevant to justify the transport of sick people or corpses, to the point of normalizing it.

*“We don’t have any concern for us as you hear your phone ringing you can be having someone calling you to carry the sick person or the dead person so at that time, I say on my own I am earning money, I don’t think about anything, but I think about money” (Boda boda driver, Kasese).*

It is worth mentioning that drivers tried to include the preventive protection measures available to them:

*“Yes, I do! [carry sick people] (…) I carry them a lot, but I encourage them wash hands and I also wash, we all put on masks and go to hospital. We have to help one another”. (Boda boda driver, Kisoro).*

The economic aspects of the need to earn revenue come to the fore again when reference is made to the clinical aspects of the disease. Significantly, however, when it is the drivers who are ill, the lack of references does not allow for an in-depth analysis of the decisions they make. On the other hand, when it is the people being transported who are ill, reference is not made to the risk that this may entail, but to income needs and the willingness to help others.

### 3.2. Transport drivers’ factors shaping mobility

This second main theme focuses on exploring those factors that may affect risk perception and mobility during the epidemic. These may be factors specific to being a driver and specifically in border areas, as well as those that may shape their perception of the disease, perceived risk, and trust.

#### 3.2.1. Associated prevalent factors

##### 3.2.1.1. Drivers’ background and work motivation

Throughout the IDIs and FGDs, the reasons for mostly young men choosing to become drivers and differences between boda boda, taxi men and truck drivers were explored. Taxi and boda boda drivers often move to urban centers in Uganda to earn money by transporting people or goods. Being able to support their families financially in a ‘masculine’ role was important.

*“I am a boda boda rider, so I also leave my home very early to come and work here in town and when I return home in the evening I am able to provide for my people, and I am also welcomed as a man.” (Boda boda driver, Hoima).*

However, many boda boda drivers and taxi drivers do not own the vehicles they drive and must pay rent to the owner. They do not have fixed salaries, and the money they make is highly variable, depending on the flow of demand. The workload of truck drivers instead, depends on material goods.

*“When cement is available, we work every day, but there are times when one can finish a full week waiting for the cement order.” (Truck driver, Kasese).*

Taxi drivers generally gather at landing sites which are established stations where drivers pick up clients whose journey may range from short distances within the town or city, to traveling to other districts or crossing international borders. For boda boda drivers, although there are established points called stages where they gather to wait for clients, they are not restricted to a particular point and the itinerary can constantly vary. Although their routes tend to be shorter, they move to urban and rural places, reaching areas inaccessible to cars.

Truck drivers, however, often travel long distances, to other districts or countries such as DRC or Kenya, according to their route plan.

*“However, the 13 hours given to us per day, we have a full hour to make stopovers for a rest. All in all, as a driver I have to have my journey plan; It's the one I follow while on a journey”. (Truck driver, Kasese).*

With a few exceptions, transport drivers carry out additional paid work to increase their income, mainly related to farming or small businesses, as driving revenues are low. All drivers claimed to work for long hours and spend their limited time-off to rest, travel to visit their family or visit places of worship. These testimonies highlight how economic activities are the ones that have the greatest weight in the daily life of the drivers, with differences between boda boda, taxi and truck drivers.

##### 3.2.1.2. The context of DRC/Uganda borders

Borders are part of the everyday lives of district residents in which the study took place. Drivers refer to borders when they talk about their home places. They associate boundaries with economic opportunities, collecting passengers from different places, even beyond international borders as one of the boda boda drivers explained:

*“Am talking about that not the real border, we have some small paths where people normally use to go to their gardens and after digging, they come back”. (Boda boda driver, Kasese).*

When there were no travel restrictions people could travel freely across the DRC/Ugandan border. Many transport drivers stated that, prior to the imposition of COVID-19 regulations, they crossed the border on a regular basis to transport passengers, cargo, visit family or make business linked to the border. This boda boda driver from Kasese recalls the trade links across the border and the impact of border closures:

*People of this community still face hunger because we were using Congo as our best country to get something to use. Our gardens are there and normally our businesses are in Congo, now we have got a big problem of closing our boundaries (Bosa boda driver, Kasese).*

Drivers, seeking to adapt to the new situation at the border, search for alternatives to the official routes. With regard to crossing through illegal channels one participant stated:

*Yes, because we have relatives in Rwanda side. (…) I have aunties across; I visit for a day or night and return. Sometimes I also get a person familiar with border shortcuts to help me, especially during this time when the border is closed” (Boda boda driver, Kisoro).*

The continued crossing of border by transport drivers does not imply that they are unaware of the associated risk of infectious diseases transmission in this cross-border area.

*“The fact that this place attracts a lot of people, we are at a high risk of contracting the infectious diseases.” (Truck driver, Kasese).*

Thus, the specificities of the transport drivers in these border districts influence decisions on movements focused mainly on socio-economic factors.

#### 3.2.2. Factors associated with Ebola and COVID-19

##### 3.2.2.1. Ebola virus disease and COVID-19 knowledge and perceptions

To what extent can disease awareness influence the movements of transport workers? In general, transport drivers had an overall understanding of EVD, including its symptoms and severity.

*“I hear Ebola kills faster than HIV/AIDS, COVID-19 and any other disease because one vomits, get diarrhea and bleeds from almost every body part, eventually the victim dies.” (Truck driver, Kasese).*

None of the drivers explicitly stated that they doubted the existence of the epidemic, yet they perceived themselves to be at low risk of EVD infection. While most were aware that it occurred primarily in DRC, opinions differed as to when the last epidemic occurred, and many considered it to be several years ago. None mentioned the more recent EVD epidemic in North Kivu in 2021.

With respect to COVID-19, perceptions of the disease differed, with some doubting its existence or believing it to be politically motivated.

*“I think government would have brought a COVID-19 survivor on TV to testify about the illness publicly. But it failed to do so. Therefore, it becomes hard to believe that this disease is real or not.” (…) Why was COVID-19 very rampant during campaigns and election period? Because currently the government is talking of only 29 Covid cases. Should we say that all this was political?” (Truck driver, Kasese).*

As this testimony reflects, the perception of the disease and the risk involved depends to a large extent on the credibility of the sources of information.

##### 3.2.2.2. Trust

The restrictions imposed because of COVID-19 pandemic, led to mistrust by some drivers in the government and their representatives at local level, as they believed these were imposed to benefit themselves. From the interviews and focus groups, it can be seen that distrust of the government is most evident in Kasese district, where a lack of resources and poverty featured recurrently in the interviews.

*“We would have trusted government, but some government workers use the COVID-19 pandemic to achieve their own desires and to create rifts among community members”. (Truck driver, Kasese).*

*“A police officer who has not built a house in this pandemic is not there. Because of the high levels of corruption on the check point and on the road”. (Truck driver, Kasese)*.

Restrictions on movement and the closure of the border have aggravated the situation, limiting trade with their neighbors.

*“People face a lot of problems, as we talk now, development in business but as per now the COVID is a problem, we were having some people coming from Congo to bring something, but now it cannot because they have locked the boundaries at the border, we are doing nothing”. (Boda boda driver, Kasese).*

In contrast to the government, health workers are trusted, as drivers felt that they were well- informed.

*“Doctors always tell us what we are suffering from and confidentiality is exercised”. (Truck driver, Kasese).*

It is therefore perceived that a lack of trust in government officials is based on abuse of their functions for financial gain, which is not the case for health workers, and the impact on drivers’ perceptions of risk of disease versus risk of reprisal for non-compliance with restrictions is explored for both epidemics.

##### 3.2.2.3. Fear

The concept of fear emerged throughout interviews in relation to perceived risk. This fear referred to the clinical consequences, including the potential fatality associated with contracting either EVD or COVID-19 or transmitting the viruses to their families.

*“After knowing that Ebola reached Congo, we all feared to go there or to meet any person coming from the side.” (Taxi driver, Kisoro).*

*“We carry different passengers who are not from the same location, but if there is a spot sensed to have a corona victim or Ebola, it becomes a big challenge to us because (…) we also have our families that we leave back home and getting in contact with someone with Ebola or COVID, we as well carry it home to our innocent families”. (Boda boda driver, Kisoro).*

Fear, however, did not deter drivers from crossing the international border to carry out their activities:

*“We used to travel while scared (…) Yes indeed (crossing the border while scared)! We could go to Goma in Congo side but really worried” (Truck driver, Kisoro)*

Nonetheless, the population-level fear appeared greater for COVID-19 compared to EVD.

*“When Ebola came, there was no lockdown and strict measures put in place. When COVID-19 came the whole country went under panic and at a standstill, in other words the COVID-19 pressure overshadowed Ebola” (Truck driver, Kasese).*

In contrast to EVD, the fear associated with COVID-19 also extended to the punishment for non- compliance with the restrictions.

*“COVID-19 affected us much. We got locked down for around three months, there was a lot of fear because army used to beat people”. (Boda boda driver, Kisoro).*

Despite the fear of diseases, the drivers emphasized their need to continue working to earn a living. This argument is emphasized by the response of one of the boda boda drivers when asked about the fear that COVID-19 and/or EVD could easily spread in the driving community.

*“We know that, but we have to survive at the same time. They also gave us only one mask, they got old, so what are we supposed to do?”. (Boda boda driver, Kasese).*

Drivers’ discourse reveals a marked difference between EVD and COVID-19. With regard to EVD, fear is associated with contracting the disease but in the case of COVID-19 an additional factor is added, fear of the consequences of non-compliance with restrictive measures. Despite this existing fear, drivers continue in some cases, to breach mobility restrictions because of the need to earn an income.

## 4. Discussion

Our research is the first to compare differences in the movement patterns and disease perceived risk from the perspective of transport drivers in three border districts of Uganda during the EVD epidemic and the COVID-19 pandemic.

Social and demographic circumstances can have a significant impact on the spread of infectious diseases but can also lead to increased vulnerability of certain sub-groups (38, 39). This reflects the need to integrate insights from different sectors and disciplines. Social science can contribute to a better understanding of infectious disease transmission in the unique context of these bordering areas and the idiosyncrasies of the drivers with the aim to understand their peculiarities and reveals the need for adapted preventive measures.

### 4.1. Uniqueness of borders in the spread of epidemics

Human mobility is intrinsically associated with the spread of infectious diseases (40). Borders between regions and countries are particularly relevant when it comes to understanding the transmission of infectious diseases. These borders are particularly relevant when communities on both sides integrate socially, economically, and politically along a porous border (7), thus increasing the risk of transmission from one area to another. Thus, borders are part of the identity of drivers living in districts close to the border between DRC and Uganda, where every day the inhabitants on both sides of the border cross it to cultivate their land, go to the market or even seeking medical care.

Border control measures are not always aligned with the needs of the communities on both sides, so the search for alternative routes is frequent. Informal border crossing between DRC and Uganda is long documented and normalized (41) and is explained to avoid procedures, taxes, or smuggling. Findings in this study are consistent with previous studies regarding the persistence of unauthorized movements regardless of ongoing epidemics (7), increasing the risk of spreading the disease to other countries.

### 4.2. Driver idiosyncrasy in disease transmission

In addition to the demographic context, social factors can significantly influence the transmission of infectious diseases, even increasing vulnerabilities in some population subgroups. As for other diseases, social groups with economic difficulties suffer a greater burden to cope with infectious diseases (38).

Findings from this study show that boda boda drivers, taxi and truck drivers share several work-related factors that put them at risk of epidemic transmission, such as EVD and COVID-19. Results from the present study are consistent with previous research about transport drivers in Uganda, identifying them as high mobility groups at risk in the transmission of epidemics, including EVD (7, 26, 33, 42, 43). Their high mobility to a wide range of destination, potentially leads to contact with a large pool of people from different places, making them vulnerable to contract communicable diseases.

However, variability in workload and low incomes, can result in economic instability. Contrary to the social image of daredevils, many transport drivers are responsible for the household finances, giving greater relevance to get revenues. This high dependency on daily income renders them vulnerable to changes that affect their livelihoods, making it difficult for them to reduce or stop working even under epidemic restrictions.

### 4.3. Divergence EVD and COVID-19

Differences in disease awareness between the EVD epidemic and COVID-19 pandemic among transport drivers, as well as different public health measures adopted led to distinct risk perceptions and differences in mobility between the two. EVD disease awareness was high, with drivers acknowledging ways of transmission, preventive measures, and high lethality. However, risk was not perceived as an imminent threat, as no cases affected their communities, and it was difficult for them to locate the epidemic in time. Moreover, none of the transport drivers referred to the epidemic that affected the neighboring DRC districts of North Kivu in 2021, probably under shadowed by COVID- 19. Also, public health measures did not entail a disruption of their daily activities for most of the drivers. Restrictive measures during the EVD epidemic were thus understood to protect people from contracting and spreading the disease.

The COVID-19 pandemic shows a different picture. Although COVID-19 monopolized the media, the changing information about COVID-19 did not translate into a clear message and the driving community was generally doubtful about the origin of the disease, the symptomatology, or the transmission of the virus. Although fear of contracting the disease was initially perceived, this was quickly shifted to a fear of retaliation by local armed forces. Drivers interpreted these restrictive measures as a means for armed personnel to gain economic benefits through the abuse of force, as they had been caught crossing the international border; driving through town after curfew; or transporting passengers on the back of a motorcycle. This led some drivers to question the existence of the pandemic.

This discourse was especially evident in Kasese district, where border agents were accused of benefiting from bribes imposed to allow movement and border crossing. A study conducted in the area shows how this distrust of the local armed forces and the government in general precedes the COVID- 19 pandemic in this area, where the armed forces were present since 2017 to prevent the spread of EVD from the DRC by imposing measures considered unfair by the population. In addition, there has long existed in the area a rebel group seeking independence from the Rwenzururu kingdom and showing open hostility toward the government and its president Museveni (44, 45).

Unlike the EVD epidemic, COVID-19 led to a disruption of global mobility and greatly impacted those working in unregulated jobs, as some of the transport drivers (46, 47). Moreover, measures aimed at reducing the risk of transmission in Uganda were among the most restrictive in the world (48) with a high impact on population mobility and particularly transport drivers. The closure of borders, the reduction or prohibition of passenger transport, curfews, and the restrictions on boda boda drivers to work after certain hours, led to a disruption of their daily lives which, although initially linked to the fear of infection, was progressively replaced by the threat to their livelihoods and the fear of reprisals from security forces. A study conducted in two Ugandan localities bordering the DRC during the COVID-19 pandemic shows how local official public authorities benefited from centrally established priorities to improve their position, while limiting the mobility of the population. This led communities to create new ways of subverting these regulations, including new forms of cross-border movements. Thus, the study argues that the impact of a militarized response was greater than that of the disease itself (45).

These differences in risk perception and fear are blurred by the imperative need to work, leading transport drivers to adopt risky practices such as disregarding regulations, working or visiting other communities even when feeling unwell and carrying sick people or dead bodies.

In this study, we argue that economic hardship leads drivers to non-compliance with regulations regarding mobility limitations, despite the fear of illness or consequences of non-compliance with mobility-restricting measures. Moreover, the acceptance of these measures is impacted by fraudulent practices by certain officials, which were mostly reported during the COVID-19 pandemic, negatively impacting distrust toward authorities.

### 4.4. Limitations and future directions

One limitation of the study was the risk of participant recall bias, as the restrictions in place at the time of the interview (May 2021) were already relaxed. This could have influenced their perception of the restrictions during the time these were tightened. At the same time, it is worth considering a similar bias as the investigation was conducted while the COVID-19 epidemic was still present with an increase in the number of cases during the time when the interviews were conducted while the EVD epidemic in DRC (2018–2020) had ended nearly a year ago.

The drivers who participated in the study reside in urban areas, where they carry out their work activities. The incorporation of drivers who reside and work in rural areas would have given a broader view of the impact of restrictions on their daily activities and consequently on perceived risk and fear. Furthermore, a medical diagnosis for COVID-19 was not included. Feeling sick corresponded to a subjective perception by participants without specifying signs or symptoms. Thus, those might include other conditions.

The selection of themes and codes was done by a single investigator (as part of a MSc dissertation), which can bias the results described above.

It would be useful to conduct future research to analyze perceptions regarding the COVID-19 pandemic once the restrictive mobility measures have been completely relaxed, thus reducing the impact on drivers’ daily activities and compare them with the findings of this study. It is worth considering that during the design and data collection period of this study, the COVID-19 vaccine was not available and therefore the views of transport drivers regarding this were not obtained. However, in future studies we would recommend including discussion about both EVD and COVID-19 vaccines in this population.

Although the districts in which the study was conducted are not among those where cases of EVD have been found during the current epidemic, the perception of restrictive measures, perceived risk, fear of the disease and trust in the authorities could shed light on the need for context-specific measures for drivers in border areas to help control transmission of disease.

As with the group of drivers, it would be interesting to include in the study people from other groups also considered vulnerable to epidemics, such as sex workers or health workers, and to compare the perceptions of the different collectives, considering the idiosyncrasies of each of them.

## 5. Conclusion

Restricting mobility has been, and still is, one of the main measures in the control of COVID-19. However, a previous study revealed that local and international travel restrictions are effective at initial stages of an epidemic. However, at later stages, behavioral changes gain relevance in containing the spread and restrictions in movements become inefficient. Measures addressing mobility and a focus on behavioral change should consider transport drivers’ reality and the context of these border areas.

Transport drivers’ movements under these regulations as seen in these findings, reinforce the argument that, despite the perception of risk and fear; the economic constraints, the responsibility to their families and social solidarity, the transport drivers continue to work.

Finally, the presence of EVD and COVID-19 does not seem to have greatly altered the transport of sick or dead bodies, as recognized in the interviews by some transport drivers, mainly boda boda drivers. Some take precautions such as asking for symptoms; however, economic needs again take precedence over risk and fear. This implies that transport drivers continue to carry passengers even if these are sick, increasing the risk of contracting COVID-19 and then infecting others.

This highlights the need to adapt preventive measures and policies in the prevention of the spread of infectious diseases to the realities of drivers in border areas.

### 5.1. Recommendations

Findings from this study provide information to policymakers for the design of effective public health policies and recommendations related to the spread of COVID-19 and future epidemics among transport drivers. We therefore recommend that:

1. Health education messages are conveyed in a clear manner, allowing transport drivers to raise and clarify questions and concerns about the disease, encouraging the sharing of opinions about risks and fears and building common solutions. These messages should be tailored to their context and realities, for example, providing health education at landing sites and stages. Communication channels and trusted message conveyors, such as health workers or local leaders, should be chosen to increase messages’ credibility.
2. Policy makers should consider how mobility restrictions affect transport drivers and impose these only when necessary. Instead, they could continue to encourage the use and provide non- pharmaceutical interventions such as face masks and sanitizers on a regular basis. The government should consider the possibility of providing financial support when mobility restrictions limit or make it impossible for drivers to work. Regulated driver associations can also manage state aid and organize fundraising to support drivers in need during COVID-19 and future epidemics.
3. Trusted local leaders and drivers’ associations should serve as a bridge between transport drivers and law enforcement officers, improving communication and understanding between them.
4. The government should monitor authorized border points and penalize those who are involved in corruption.
5. Continue to allow cross-border workers when possible, and increase measures aimed at screening and IPC, discouraging the use of unauthorized border points.

For these proposals to be appropriate and effective, local leaders, health teams and transporters’ representatives must be involved and participate in the design of health policies affecting transport drivers.

Border communities are highly connected for a variety of social and economic reasons. These daily realities are in direct conflict with guidance to limit travel during an active Ebola epidemic and the COVID-19 pandemic. Social science research allows us to understand the realities of the communities on both sides of the border, as is the case of transport drivers. Policy makers should consider these specificities when establishing public health measures and recommendations affecting mobility during COVID-19 and future epidemics.

## Data availability statement

The datasets presented in this article are not readily available because the participants of this study did not give written consent for their data to be shared publicly, so due to the sensitive nature of the research supporting data is not available. Requests to access the datasets should be directed to Shelley.Lees@lshtm.ac.uk.

## Ethics statement

Ethical approval was obtained from the London School of Hygiene and Tropical Medicine (Reference number 25417). The main study of which this research is part was approved by LSHTM Ethics committee (reference number 17860), the Makerere University School of Social Sciences, Research Ethics Committee (reference MASSK.REC.0819.330), and the Uganda National Council for Science and Technology (reference SS). The patients/participants provided their written informed consent to participate in this study.

## Author contributions

MB-P: study design, data analysis, and manuscript writing and revision. HB, AB, SC, and CF: study design and manuscript revision. MS-S and DK-M: study design, data collection and manuscript revision. CI, HM, JR, and GNM: data collection and manuscript revision. SL: study design and manuscript writing. All authors contributed to the article and approved the submitted version.

## Funding

This research is funded by the Centre for Disease Control and Prevention (CDC) and UNICEF. HB was funded by the Department of Health and Social Care using UK Aid Funding as part of the UK Vaccine Network and is managed by the National Institute for Health and Care Research.

## Conflict of interest

The authors declare that the research was conducted in the absence of any commercial or financial relationships that could be construed as a potential conflict of interest.

## Publisher’s note

All claims expressed in this article are solely those of the authors and do not necessarily represent those of their affiliated organizations, or those of the publisher, the editors and the reviewers. Any product that may be evaluated in this article, or claim that may be made by its manufacturer, is not guaranteed or endorsed by the publisher.

## Author disclaimer

The views expressed in this publication are those of the author(s) and not necessarily those of the Department of Health and Social Care.
